# Ordinary Infant Dietaries

**Published:** 1911-04-01

**Authors:** 


### ORDINARY INFANT DIETARIES.

A great deal of the illness among the infants and small children of the poor is due to errors of dieting or to improper care of food that otherwise might be good, particularly during the summer season. The proper food for an infant is its mother's milk. If the mother has plenty of breast-milk no other food should be given. The infant should be suckled as follows: During the first two months, every 2 hours; during the third month, every 2½ hours; during the fourth month and onwards, every 3 hours.

Infants should be suckled regularly by day but not disturbed at night. If the infant is asleep when a night-feed falls due, the feed should be omitted.

If the mother has not sufficient breast-milk, then she should take additional fluid, preferably not any alcoholic beverage, such as stout, which is so often recommended, but rather a large cup of milk or milk and warm water half an hour before each nursing. If there is still insufficient breast-milk to satisfy the child, the latter should not at once be weaned, but it should be given additional feeds from a bottle, the composition of these extra feeds varying with the age of the infant as described below. If the child must be brought up by hand it should be fed on cow's milk mixed with water, warmed and sweetened, with a little sugar. Barley water may be used instead of plain water if the latter does not seem to suit, the method of making barley water being to take one teaspoonful of prepared barley, powdered, adding it to one pint of boiling water, boiling for five minutes, and then straining through fine muslin or a fine sieve.

All town milk, as soon as it is received into the house, should be boiled for one minute and then stored in a cool place in a vessel that is kept closely covered.

The milk and the water or barley water should be mixed in the following proportions according to the infant's age:—

| Age of Infant | Amount of Milk | Amount of Water or of Barley Water | Total Amount to be Given at Each Feed |
|---|---|---|---|
| | Tablespoonfuls | Tablespoonfuls | Tablespoonfuls |
| 1st and 2nd week ... | 1 ... | 2 ... | 3 |
| 3rd and 4th week ... | 2 ... | 4 ... | 6 |
| 2nd month ... ... | 3 ... | 5 ... | 8 |
| 3rd month ... ... | 4 ... | 5 ... | 9 |
| 4th month ... ... | 6 ... | 6 ... | 12 |
| 5th and 6th month ... | 8 ... | 6 ... | 14 |
| 7th to 9th month ... | 16 ... | None ... | 16 |

The amount of sugar that should be added is about one teaspoonful for every eight ounces of mixture.

Boat-shaped bottles without tubes should be used. They should be thoroughly rinsed out after each feed, and kept submerged in a basin of clean cold water. Each bottle and teat should also be thoroughly scalded at least once a day.

No gruel, bread, nor any kind of solid food should be given to a baby under eight months old. From eight months to one year of age the child should still be chiefly fed on milk but should have in addition bread and milk, and milk thickened with baked flour or with some kind of prepared infants' food. From one to two years of age the child should be fed on bread and milk, bread and gravy, broth, bread and butter, mashed potatoes, lightly boiled eggs, milk puddings, and cow's milk.

